# The use of a rein tension device to compare different training methods for neck flexion in base‐level trained Warmblood horses at the walk

**DOI:** 10.1111/evj.12831

**Published:** 2018-04-06

**Authors:** I. Veen, D. Killian, L. Vlaminck, J. C. M. Vernooij, W. Back

**Affiliations:** ^1^ Department of Equine Sciences Faculty of Veterinary Medicine Utrecht University Utrecht The Netherlands; ^2^ Faculty of Science Utrecht University Utrecht The Netherlands; ^3^ Department of Surgery and Anaesthesiology of Domestic Animals Faculty of Veterinary Medicine Ghent University Merelbeke Belgium; ^4^ Department of Farm Animal Health Faculty of Veterinary Medicine Utrecht University Utrecht The Netherlands

**Keywords:** horse, rollkur, head and neck position, hyperflexion, rein tension, draw reins, auxiliary aid

## Abstract

**Background:**

Debate surrounds the use of high rein tension for obtaining different head and neck positions in the training of sport horses on account of possible welfare issues.

**Objectives:**

To compare auxiliary rein tension in two methods (Draw Reins and Concord Leader) for obtaining a standardised head and neck position on a hard and a soft surface.

**Study design:**

Intervention study.

**Methods:**

Left and right rein tensions were measured in 11 base‐level trained client‐owned sport horses (mean age ± s.d.; 10 ± 3.2 years) exercised in‐hand with, in a random order, conventional draw reins or the newly developed Concord Leader in a standardised head and neck position. Rein tension was measured using a calibrated device operating at 10 Hz during six runs of 15 s in a straight line for each training method on both a hard and a soft surface. A linear mixed model and grouped logistic regression analysis were applied to compare the two methods (P<0.05).

**Results:**

The odds of a tension of 0 N were lower with draw reins than with the Concord Leader. The rein tension (mean sum of the force applied, in N) of the draw reins was 13.8 times higher than that of the Concord Leader.

**Main limitations:**

This study was performed on horses exercised in‐hand; however, these auxiliary aids are normally used when lungeing. Possible redirection of rein tension towards the poll was not measured.

**Conclusions:**

We showed that when using the Concord Leader a similar head and neck position is achieved with a much lower rein tension than with the draw reins and, more importantly, with a much greater likelihood of 0 N. It is unnecessary to use high auxiliary rein tension to obtain a standard, flexed head and neck position.

## Introduction

During training and competition, discomfort in horses may be poorly recognised by owners and caretakers and sometimes even inflicted by them 1. Tension applied to the reins creates pressure on oral tissues 2, 3, 4, 5 and several types of pathology are associated with this 6, 7, 8, 9, 10, 11, 12. Training horses involves the use of negative reinforcement whereby applied rein tension is released when the horse responds in the desired way 13, 14 but this may lead to habituation to rein tension 13, 15, 16.

Nowadays, the desired head and neck positions used in training and competition might be different from what they were a decade ago, as Lashley *et al*. 17 found a difference in head angle of the horses between top‐level dressage competitions in 1992 and 2008 suggesting changes in head and neck positions over the years. Since the head and neck position significantly influences the kinematics of the horse 18, 19, 20, 21, the use of different head and neck positions may be a risk in terms of the welfare of the horse. In particular the extremely flexed head and neck position (hyperflexion/rollkur) has a wide range of mental and physical effects on the horse 6, 22, 23, 24, 25, 26, 27, 28, 29.

**Figure 1:**
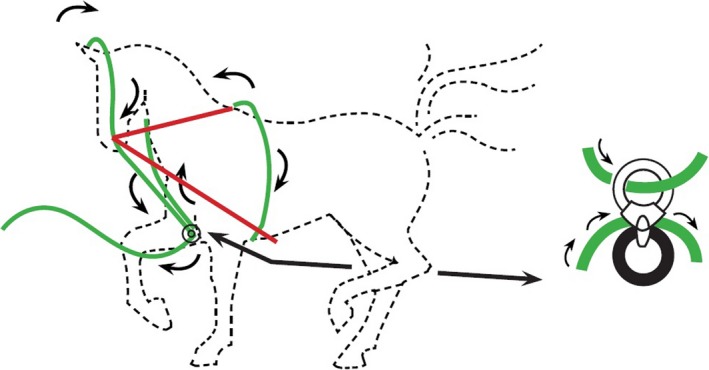
Schematic reproduction of the working mechanism of the draw reins (red) and Concord Leader (green).

Modern techniques of asking the horse to be ‘on the bit’ usually require the horse to change the angle of its neck to find relief from pressure applied by the rider through the bit and reins 13, thereby positioning the nose behind the vertical line 17, 30. There is much discussion in the equine world about riders using high rein tension to obtain a desired head and neck position. Additional means, such as draw reins, are also used to obtain a desired head and neck position 14, 31. Because of the welfare issues associated with high rein tension, we measured the rein tension necessary for obtaining a desired head and neck position with two auxiliary aids on a hard and a soft surface; Draw reins and Concord Leader^a^. Draw reins are a confined method for obtaining a desired head and neck position because the system has two fixed points (Fig 1). The Concord Leader is not as restricted, as it functions as a continuous loop, works with a pulley, and has no fixed points (Fig 1).

Horses have a shortened stride duration on a hard surface compared with a softer surface 32. As limb kinematics could influence rein tension we expected a difference in rein tension on the different surfaces.

## Materials and methods

### Horses

We used 11 client‐owned Warmblood sport horses (three mares and eight geldings) that were base‐level trained. The horses were familiar with but not habituated to the draw reins and the Concord Leader; they were walked twice using both methods prior to the study and horses trained with one or both of the methods were excluded. Their mean age (±standard deviation [s.d.]) was 10 ± 3.2 years and their mean height (±s.d.) was 168.9 ± 4.5 cm at the time of measurement.

### Draw reins and Concord Leader

The head and neck position used in this study was as follows: the bridge of the nose was around the vertical and the rostral part of the mouth was at the level of the supraglenoid tubercle (HNP2 22). The draw reins and Concord Leader were fitted in standstill so that the horse was in this head and neck position at the start of the measurement (Supplementary Items 1 and 2). The draw reins were attached to the dorsal side of the surcingle and ran through the bit back to the ventral side of the surcingle (Supplementary Item 1). The Concord Leader was fitted around the thorax just behind the withers and through the forelimbs up to and through the right bit ring, over the head (behind the ears) and through the left bit ring back to the pulley between the forelimbs and then to the handler (Supplementary Item 2). To prevent the handler from influencing our standardised head and neck position the ropes were knotted, so that the hand of the handler adopted a relatively fixed position (Supplementary Item 3).

### Rein Tension Device

The Rein Tension Device, which was developed at Utrecht University (Utrecht, The Netherlands) recorded at 10 Hz. The measurement range of the Rein Tension Device is 0–200 N and the resolution is 10 bits. According to the Nyquist sampling theorem, 10 Hz measurements are within the criteria for the walk, because the frequency of changes in rein tension due to the stride cycle will be lower than 2.5–3 Hz 33. Before the start of each measurement the horse was in standstill and the reins were hanging loose; therefore, the only tension in the reins was that of the weight of the sensors and reins, under which conditions the device was calibrated to zero. The Rein Tension Device is based on a linear, solid state force transducer (Flexiforce)^b^ and converts force to resistance. This linear transducer was read by a linear electronic circuit that converted resistance to N (units of force). Given the linear nature of the system, the system was calibrated by applying two different weights (calibrated weights of 1 and 2 kg) to the transducers while measuring the output signal in V. These measured values were used to calculate the slope and intercept of the transfer function. This was performed separately for both transducers of the tension meter. The values of the slope and intercept were used in the software of the Rein Tension Device to calculate the force on each transducer. The reproducibility of the measurements was validated by applying the same weights to the transducers and comparing the outcome with the original values, which were identical. The sensor of the Rein Tension Device was attached to the bit and to the draw reins or Concord Leader. The cables from the Rein Tension Device ran from the sensors to the device itself. The device was attached to a surcingle, which was fitted to the horse. The bridle and bit were fitted to the horse according to the following criteria:

• The throat lash was fitted so that an upright fist could be inserted between the mandible and the lash.
• The noseband was fitted so that two fingers could be inserted between the bridge of the nose and the noseband.
• The lower noseband was fitted so that two fingers could be inserted between the nose (at the level of the incisura naso incisiva) and the lower noseband.
• The bit was fitted so that there was only one fold of skin in the corner of the mouth and the bit extended from the mouth by the width of one finger or less. Identical double‐jointed snaffle bits in different sizes (12.5 and 13.5 cm) were used for the study; these bits have been classified as comfortable 12.

### Data collection

The horses were exercised in‐hand on a straight line (run) and rein tension was measured continuously. The same person walked, on the left side, with the horses during the whole study. A lead rope attached to the noseband was used to guide the horses Supplementary Item 3). The sequence in which the horses walked with the different methods and on the different surfaces was allocated at random. Each horse walked six runs of 25 m, within a time limit (14.5–15.5 s), with each method and on each surface. The hard surface was an asphalt trot up area and the soft surface was an indoor footing system (Agterberg Geofibremix)^c^. There were four conditions: draw reins/hard surface; draw reins/soft surface; Concord Leader/hard surface; Concord Leader/soft surface. The walking speed of the horses was thereby standardised at between 1.6 and 1.7 m/s.

Each run was filmed at an angle of approximately 90° to ensure a good view of the start and finish of the run as well as the movements of the horses. Rein tension was continuously (10 Hz) measured and using this film footage we were able to connect the rein tension measurements to the specific runs and thereby ensure that the appropriate data were selected.

### Data analysis

The data were stored on a memory card in a text file and then converted into a custom‐made spreadsheet (Microsoft Excel 2007)^d^. For extraction of the correct data, the film was analysed using commercial software (Adobe Premiere Pro CS 7)^e^.

The spreadsheet was used to extract the correct data for import into the statistical program R 34. For each horse 150 values (N) per condition and side were obtained. The values were summarised by calculating the sum of the values (sum of tension) and obtaining the frequency of measurements with 0 N values respectively for the left and right side separately. This resulted in 48 summarised observations (four conditions × two sides × six runs) for both outcomes per horse. The Pearson's correlation coefficient was calculated of the sum of the tension values per run between the left and the right rein on the same run.

The number of 0 N values per run was analysed using grouped logistic regression analysis (the frequency of 0 N values in a total of 150 measurements), using the horse as the random intercept to account for repeated measures within a horse 35. The explanatory factors were: side; method and surface; and the interaction between method and surface.

The outcome (sum of the tension) per run was analysed using a linear mixed model with the horse as the random intercept to account for repeated measures within a horse 36. The explanatory variables were side, rein method and surface and the interaction between rein method and surface. A constant variance function was added to allow the data to have different variances per method. The outcome variable (sum of the tension) per run was log transformed (log(sum+1)) to meet the model assumption of normality. The model assumptions were checked by visual inspection of the residuals on a Q–Q plot and scatter plots of residuals vs. the respective three factors and predicted values. Profile likelihood 95% confidence intervals (CIs) for the estimated parameters were calculated. For both outcome variables the Akaike information criterion (AIC; between competing models the model with lower value is better) was used to select the best model using a backwards selection procedure 37.

## Results

**Figure 2:**
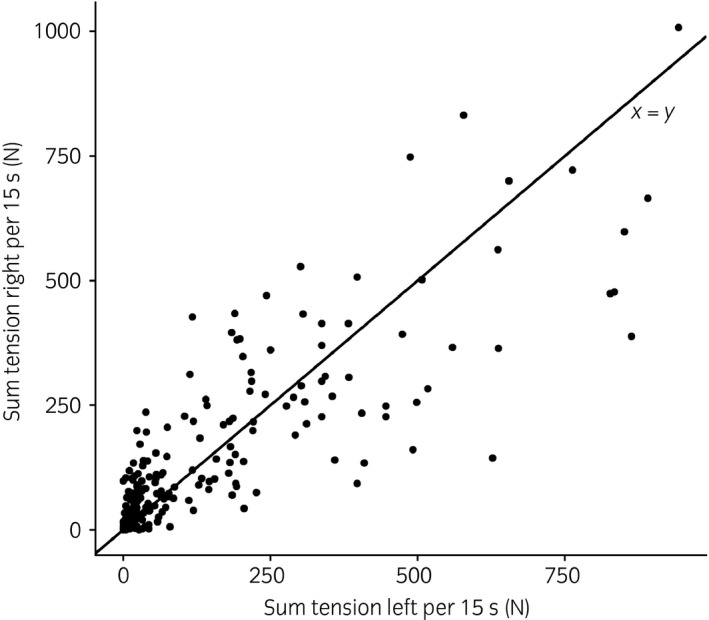
Scatterplot between rein tension (measured as the sum of Newton values) in the right rein (y‐axis) and rein tension in the left rein (x‐axis) per run (15 s). The x = y diagonal is given.

Supplementary items 4 and 5 give a graphical impression of the raw data (full 15 s and 1 s interval respectively) as they were collected during the measurements. There was a positive correlation (r^2^ = 0.71, P<0.001) between the sum of tension per run in the right rein and in the left rein showing in general lower sum of tension values on the left side (Fig 2).

**Figure 3:**
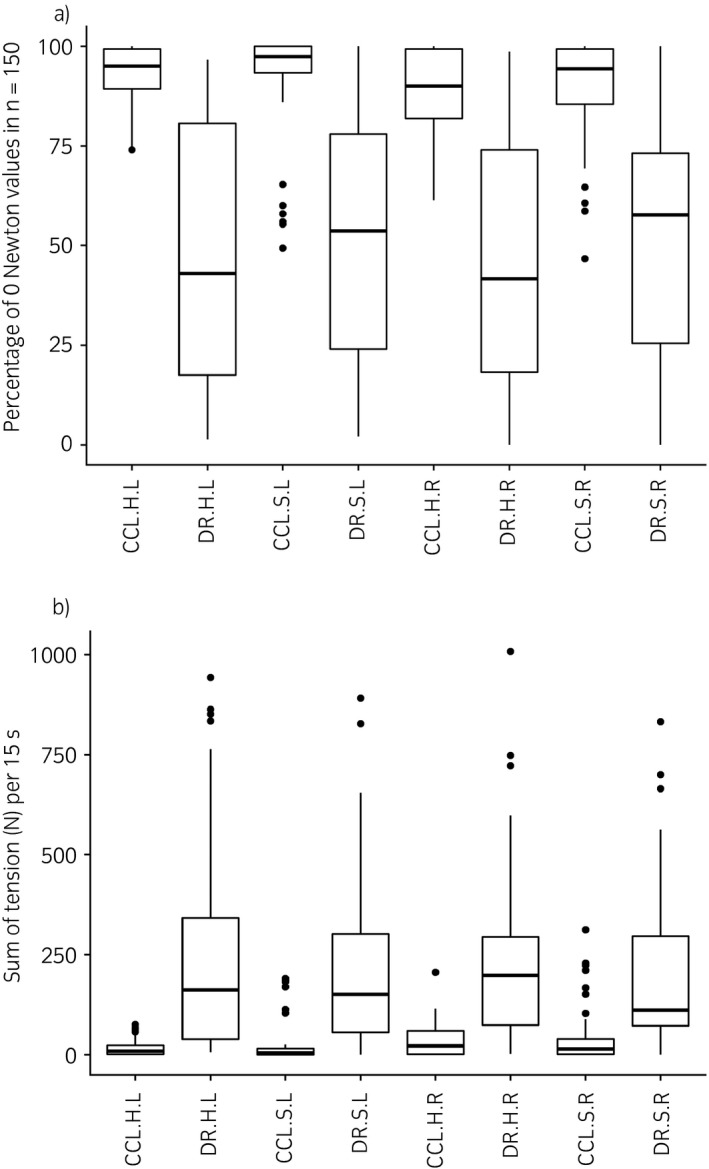
Percentage of achieving 0 Newton per run (15 s) per condition (a) and rein tension measured as the sum of Newton values per run (15 s) per condition (b). CCL, Concord Leader; DR, draw reins; H, hard surface; S, soft surface; L, left rein and R, right rein. CCL.H.L, concord leader hard surface left rein.

Figure 3a shows the percentage of 0 N values in 150 measurements per method, surface and side. The Concord Leader has, on average, a higher percentage of 0 N with smaller variation. The odds of achieving 0 N were lower with draw reins compared with the Concord Leader (Table 1). The right rein tension had lower odds of 0 N values than the left rein. Surface had no influence on the odds of achieving a tension of 0 N with the Concord Leader but affected the odds of 0 N with draw reins. The hard surface was less likely to achieve 0 N with draw reins than the soft surface.

**Table 1:** Estimated odds ratios for the frequency of 0 Newton values per run of 15 seconds using a grouped mixed effects logistic regression model^a^ under various conditions of surface, method and rein side

	Odds ratio	95% CI	P‐value
Method concord leader (ref) with hard surface	1	–	
Method draw reins with hard surface	0.04	0.038–0.043	P<0.001
Side left (ref)	1	–	
Side right	0.75	0.72–0.78	P<0.001
Hard surface with Concord Leader (ref)	1	–	
Soft surface with Concord Leader	1.1	0.99–1.15	P<0.1
Hard surface with draw reins (ref)	1	–	
Soft surface with draw reins	1.3	1.28–1.41	P<0.001

95% CI, 95% confidence interval; (ref), reference.

a The final model contains the main effects for method, surface, side and the interaction between method and surface.

**Figure 4:**
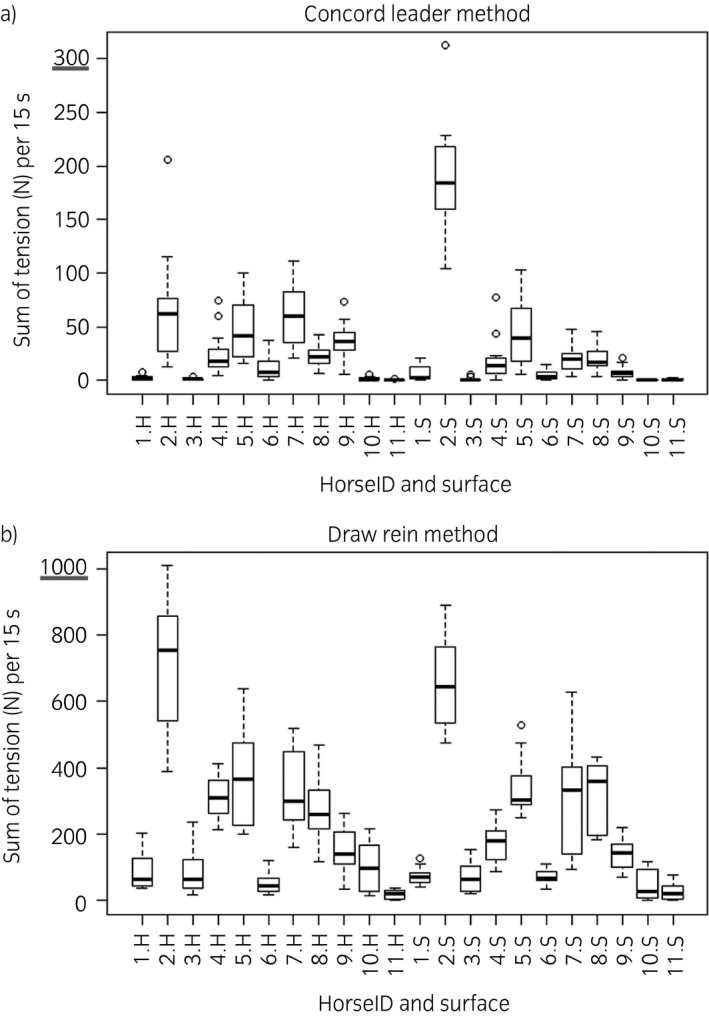
Rein tension measured as the sum of Newton values per run (15 s) per horse and surface with the Concord Leader (a) and with the draw reins (b). Please notice the different scale of the y‐axis among (a) and (b). H, hard surface; S, soft surface. 1.H, horse number 1 on the hard surface.

Figure 3b shows per‐condition box plots of the sum of tension per run. The draw reins have, on average, a higher sum of tension than the Concord Leader independent of the other conditions. Figure 4a and b are box plots of the sum of tension per horse and per surface for both methods. These box plots show considerable individual variation between horses but the sum of tension with the draw reins is, on average, higher than with the Concord Leader (on both surfaces). The mean sum of tension per run of the draw reins was 13.8 (95% CI 12.1, 15.8, P = 0.05) times higher than that of the Concord Leader. The interaction between the method and surface had no significant effect on rein tension in a run. The mean sum of tension on the soft surface was lower than that on the hard surface (estimate 0.82, 95% CI 0.72, 0.94, P = 0.05) and in the right rein the sum of tension was 1.3 (95% CI 1.1, 1.5, P = 0.05) times higher than in the left rein. Box plots of the sum of tension per horse and method on both surfaces are available in Supplementary items 6 and 7.

## Discussion

In this study, rein tension was evaluated using two auxiliary rein methods to obtain a standard, flexed head and neck position in horses exercised in hand, namely conventional draw reins and the newly developed Concord Leader. This study showed that the Concord Leader achieves a similar head and neck position with a lower sum of tension than the draw reins. The mean rein tension is greatly influenced by the amount of 0 N and that is why the analysis of the frequency and likelihood of 0 N was also performed. This study showed that the Concord Leader achieves a similar head and neck position with a greater likelihood of applying 0 N. Because of the greater likelihood of 0 N values evident with the Concord Leader, it may be useful in the training of horses.

Optimal rein tension, however, remains unclear. Rein tension is dependent on many factors, including the rider, riding discipline, level of training of both horse and rider and the gait. Young, tension‐naïve horses apply a surprisingly high rein tension (up to 40 N) to obtain a food reward in a voluntary test situation 15. Absolute rein tension values reportedly range from 2 to 104 N 38. The overall mean rein tension recorded in a long‐reining test was 10.7 ± 1 N 39, whereas the overall mean rein tension recorded in a riding test was 7.4 ± 0.7 N 40. In this study the mean rein tension with the Concord Leader was 0.17 N and 0.19 N on the hard and soft surface respectively. The mean rein tension with the draw reins was 1.53 N and 1.35 N on the hard and soft surface respectively. Maximal rein tension with the Concord Leader was 16 N and 47 N with the draw reins (independent of surface).

The measurements of rein tension obtained in these previously mentioned studies are incomparable because the studies are very different in their design. When riding, a certain amount of baseline rein tension is applied by the rider and the between‐rider variability and laterality of the rider also have an influence on rein tension 41, 42. This study was (for standardisation purposes) performed in horses exercised in hand; however, these auxiliary aids are normally used when lungeing. The rein tension differs between auxiliary aids and handheld reins and also when using auxiliary aids redirection of tension to other parts of the horse is possible. Horses are thought to aim for the lowest amount of rein tension possible 15 and therefore we would expect the auxiliary rein tension to be zero or as close to zero as possible.

Rein tension fluctuates considerably over the stride cycle 2, 41, 43 and also differs between the gaits 2. Horses have a larger range of head‐neck motion at the walk in comparison with the trot 18, and therefore fixation of the head and neck impedes the natural movement at the walk more than at the trot 18, 44. Furthermore, for each rein type, the minimal, maximal and mean tensions were higher with shorter reins, probably because shorter reins cause greater restriction of head and neck movement 16, 43. Measuring maximal rein tension would be more reliable with higher Hz because at 10 Hz we might miss the actual peak of maximal rein tension. Most studies measuring rein tension use a higher sampling frequency and therefore it is possible that we have under sampled data. However, the analysis we made was possible from this data.

A clear between‐horse variation in rein tension has been reported 41, 42 and apparently each horse applies different, individually comfortable, rein tension values 45. Further discussion is available in the supporting information. Within the eleven horses in this study each horse showed a tendency of lower tension with the Concord Leader compared with the draw reins, but it is acknowledged that this study is limited to eleven horses and the conclusions may therefore not apply to all horses.

Horses, like humans, exhibit laterality and this plays a role in rein tension 41, 46, 47. Purportedly, this laterality causes unequal left and right rein tension 4. Egenvall *et al*. 46 found a tendency for higher tension in the right rein and proposed that this may be a consequence of laterality of the horse and/or rider. One of the goals of training is to develop the straightness of the horse 38, 41. Although the method used for obtaining a desired head and neck position had the most influence on achieving 0 N values, the side had a small influence. In this study, the odds of achieving 0 N values were greater with the left rein than the right rein. In addition, the mean tension in the right rein was higher than that in the left. We did not focus on the dissimilarity (through correlation) between the left and right rein tensions but the results are indicative of inequality. In this study, the horses were exercised in‐hand and, therefore, the effect of the rider was eliminated. During measurement, the handler walked only on the left side of the horse and this may have influenced the rein tension, thereby explaining the difference between the left and right reins. While making the measurements, we attached a rope to the noseband to avoid increasing rein tension by pulling on the bit accidentally and, additionally, the horses were never encouraged to walk faster by pulling on the rope.

Research on the effect of surface on rein tension is scarce. We do know that the type of surface influences the stress on the bones, ligaments and tendons of the limbs and the risk of injury in trotters is higher on a hard track than on a soft track 48. Dissimilarities in limb kinematics between surfaces 32 can influence rein tension. In this study, the mean rein tension on the hard surface was larger than that on soft surface. The effect of the surface on rein tension differed between the Concord Leader and draw reins. Surface had no influence on the likelihood of a 0 N value with the Concord Leader. However, the more restricted draw reins had a lower likelihood of achieving 0 N on the hard surface than on the soft surface. Perhaps the horses found it more difficult to achieve 0 N on the hard surface than on the soft surface with the draw reins. Whether this finding is valid or not is unclear, and further research on the effect of surface on head and neck positions and rein tension is necessary.

The dynamic working mechanism of the Concord Leader provided a challenge for the standardisation of the head and neck position. Normally, the handler has some scope to move their hand, thereby influencing the rein tension: if the handler feels high rein tension, they can choose to relax the position of their hand to decrease it. This may have led to higher rein tensions when walking with the Concord Leader in our study compared with normal use of the Concord Leader. The use of the Rein Tension Device may also have had an influence. The Rein Tension Device was attached between the bit and the draw reins or Concord Leader, thereby changing the angles used to obtain the desired head and neck position. It must be recognised that rein tension does not equal bit pressure 30. In further studies, it would be a useful addition to measure the pressure on the poll, also to check whether any of the rein tension is redirected towards the poll when using the Concord Leader. Because it is not possible to discriminate between rein tension resulting from neck extension from the horse and that exerted by the rider 40, it will also be impossible to distinguish between the horse evading rein tension and low rein tension.

The method used (i.e. Concord Leader or draw reins) to achieve the desired head and neck position had the largest influence on the likelihood of achieving a rein tension of 0 N. Surface and side were of less importance. Therefore, our results suggest that to achieve low rein tension, the nature of the training method used is more important than the surface. In conclusion, this study showed that, when using the Concord Leader, a similar head and neck position is achieved with a lower rein tension than with the draw reins and it is unnecessary to use high rein tension to obtain a standard, flexed head and neck position. This study may contribute to the development of evidence‐based gymnastic training methods.

## Authors’ declaration of interests

No competing interests have been declared.

## Ethical animal research

The horses were used in this experiment with written permission from the owners.

## Source of funding

None.

## Authorship

I. Veen contributed to study design, study execution, data analysis and interpretation, and preparation of the manuscript. D. Killian contributed to study design and study execution. L. Vlaminck contributed to study design and preparation of the manuscript. J. C. M. Vernooij contributed to study design, data analysis and interpretation, and preparation of the manuscript. W. Back contributed to study design, data analysis and interpretation, and preparation of the manuscript. All authors gave their final approval of the manuscript.

## Ethical guidelines

The paper is original, has not been submitted or published elsewhere, and has the approval of all authors.

## Supporting information


**Supplementary Item 1:** Head and neck position using the draw reins.

**Supplementary Item 2:** Head and neck position using the Concord Leader.

**Supplementary Item 3**: The ropes were knotted to minimise the impact of the handler.

**Supplementary Item 4:** Raw data.

**Supplementary Item 5:** Raw data.

**Supplementary Item 6**: Summary data.

**Supplementary Item 7:** Summary data.

**Supplementary Item 8**: Rein tension per horse: Concord Leader Hard Surface Left Rein.

**Supplementary Item 9:** Rein tension per horse: Concord Leader Hard Surface Right Rein.

**Supplementary Item 10:** Rein tension per horse: Concord Leader Soft Surface Left Rein.

**Supplementary Item 11**: Rein tension per horse: Concord Leader Soft Surface Right Rein.

**Supplementary Item 12:** Rein tension per horse: Draw Reins Hard Surface Left Rein.

**Supplementary Item 13:** Rein tension per horse: Draw Reins Hard Surface Right Rein.

**Supplementary Item 14:** Rein tension per horse: Draw Reins Soft Surface Left Rein.

**Supplementary Item 15:** Rein tension per horse: Draw Reins Soft Surface Right Rein.

## Manufacturers' addresses

1 Verdonk Innovations, Huizen, The Netherlands.

2 Tekscan Inc., Boston, Massachusetts, USA.

3 Agterberg B.V., De Bilt, The Netherlands.

4 Microsoft Corp., Redmond, Washington, USA.

5 Adobe Systems Inc., San Jose, California, USA.
