# Supplementary figures and images for: The use of a rein tension device to compare different training methods for neck flexion in base‐level trained Warmblood horses at the walk
Source: Equine Vet J. 2018 Apr 6;50(6):825–30. doi: 10.1111/evj.12831 (PMC6174990; doi:10.1111/evj.12831)

**Supplementary Item 1:** Head and neck position using Draw Reins.

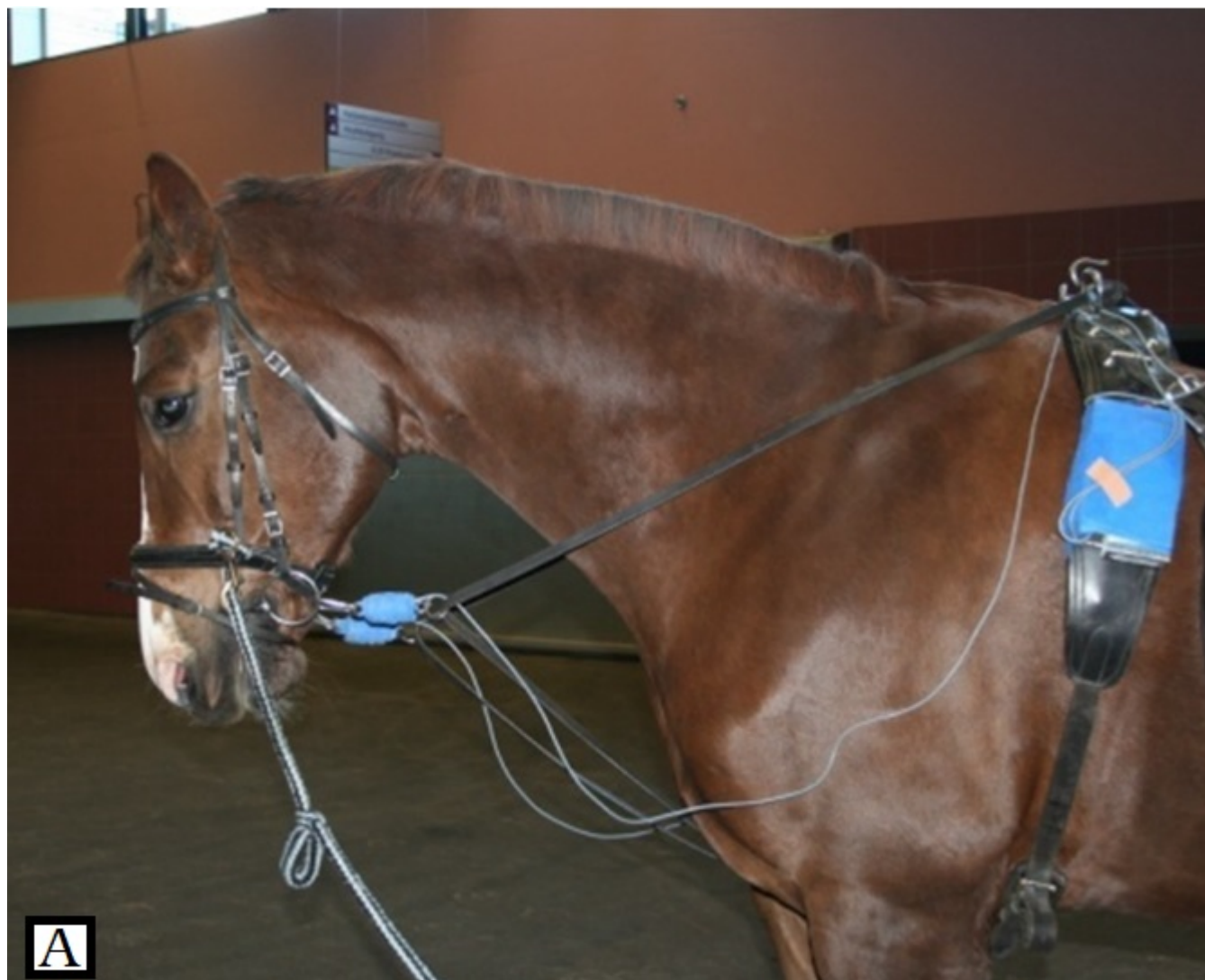

Supplement: Supplementary file 1 — Supplementary Item 1: Head and neck position using the draw reins. [file EVJ-50-825-s001.pdf]

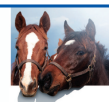

**Supplementary Item 2:** Head and neck position using a Concord Leader.

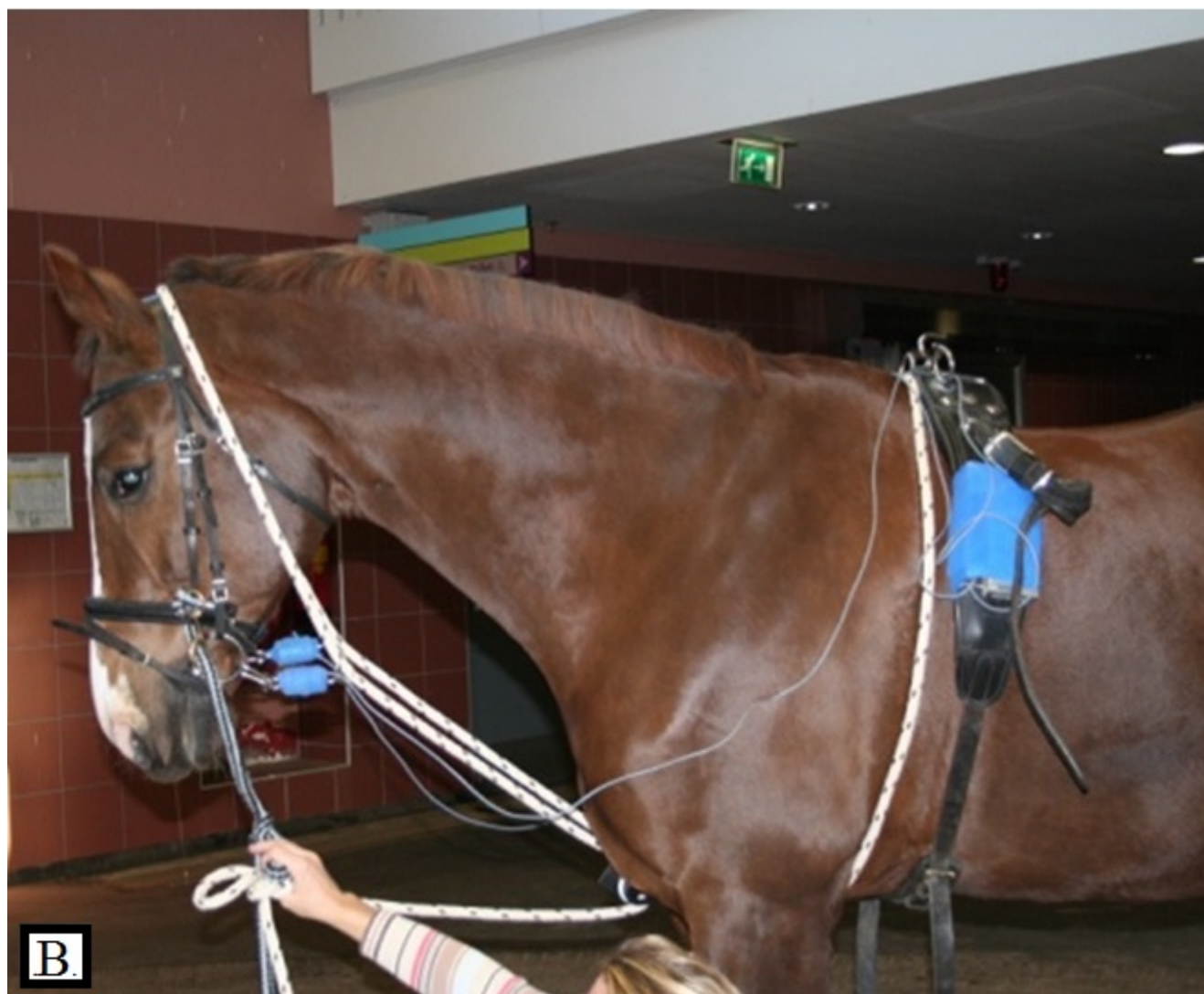

Supplement: Supplementary file 2 — Supplementary Item 2: Head and neck position using the Concord Leader. [file EVJ-50-825-s002.pdf]
