# Supplementary material for: The use of a rein tension device to compare different training methods for neck flexion in base‐level trained Warmblood horses at the walk
Source: Equine Vet J. 2018 Apr 6;50(6):825–30. doi: 10.1111/evj.12831 (PMC6174990; doi:10.1111/evj.12831)

Equine Veterinary Journal

Supplementary Item 3: The ropes of the Concord Leader were knotted to minimise the impact of the handler.

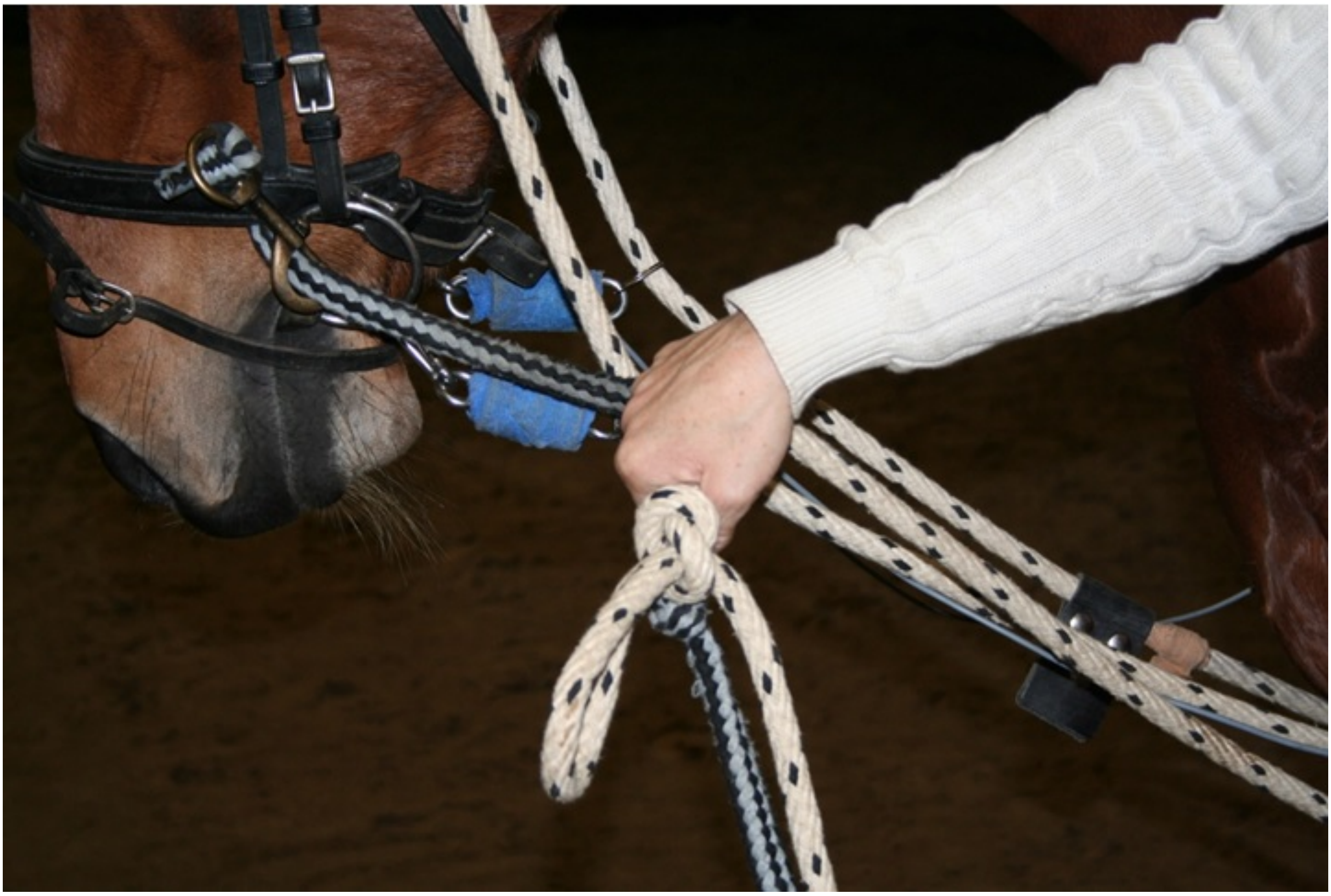

Supplement: Supplementary file 3 — Supplementary Item 3: The ropes were knotted to minimise the impact of the handler. [file EVJ-50-825-s003.pdf]
