# Supplementary material for: The use of a rein tension device to compare different training methods for neck flexion in base‐level trained Warmblood horses at the walk
Source: Equine Vet J. 2018 Apr 6;50(6):825–30. doi: 10.1111/evj.12831 (PMC6174990; doi:10.1111/evj.12831)

Equine Veterinary Journal

Supplementary Item 5: Raw data: 1 sec of rein tension Concord Leader (CCL) and Draw reins (DR) on a hard surface in the left and right rein (horse number 2).

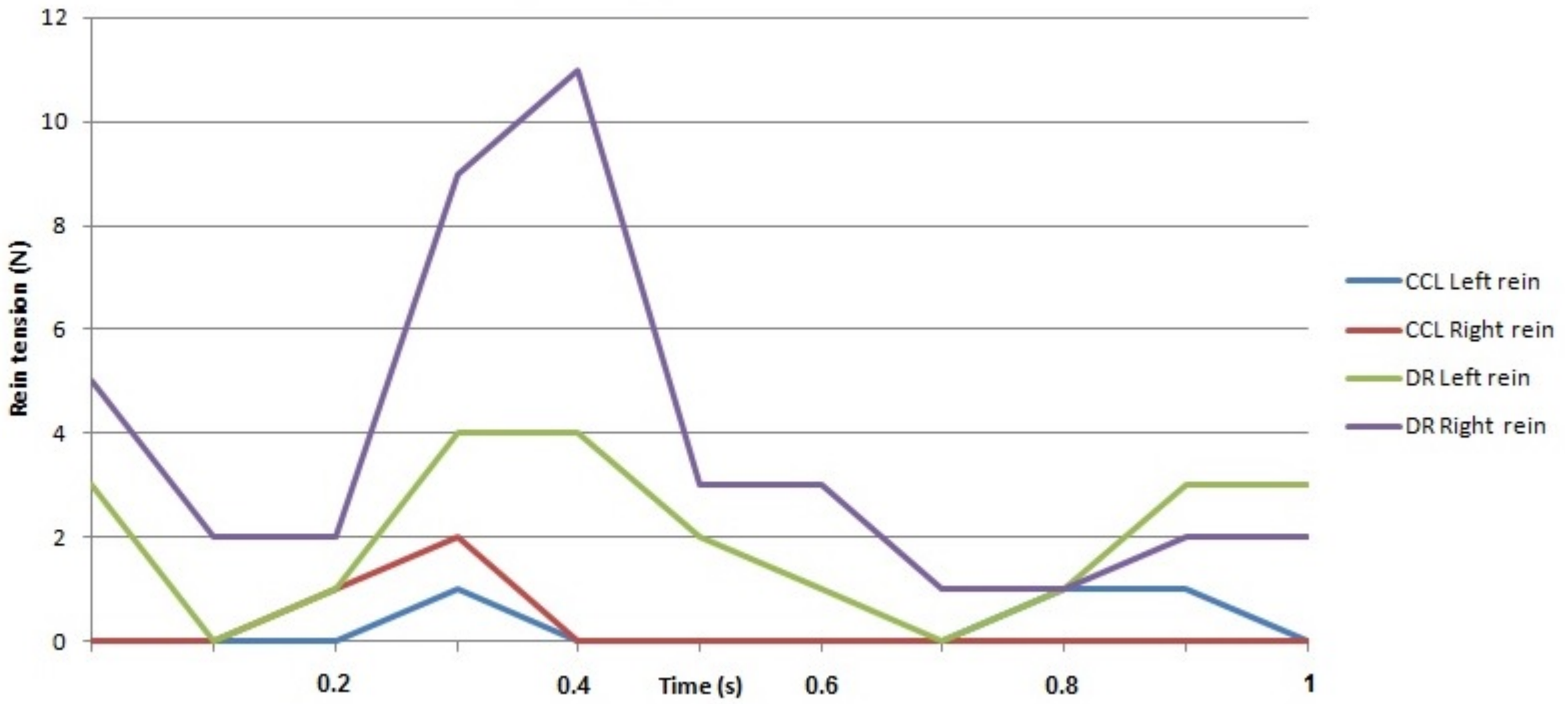

Supplement: Supplementary file 5 — Supplementary Item 5: Raw data. [file EVJ-50-825-s005.pdf]
