# Supplementary material for: The use of a rein tension device to compare different training methods for neck flexion in base‐level trained Warmblood horses at the walk
Source: Equine Vet J. 2018 Apr 6;50(6):825–30. doi: 10.1111/evj.12831 (PMC6174990; doi:10.1111/evj.12831)

Equine Veterinary Journal

Supplementary Item 7: Summary data: Rein tension measured as the sum of N values per run (15 sec) per horse and method on a soft surface.
CCL = Concord Leader; DR = draw reins. 1.CCL = horse number 1 with the Concord Leader on the soft surface.

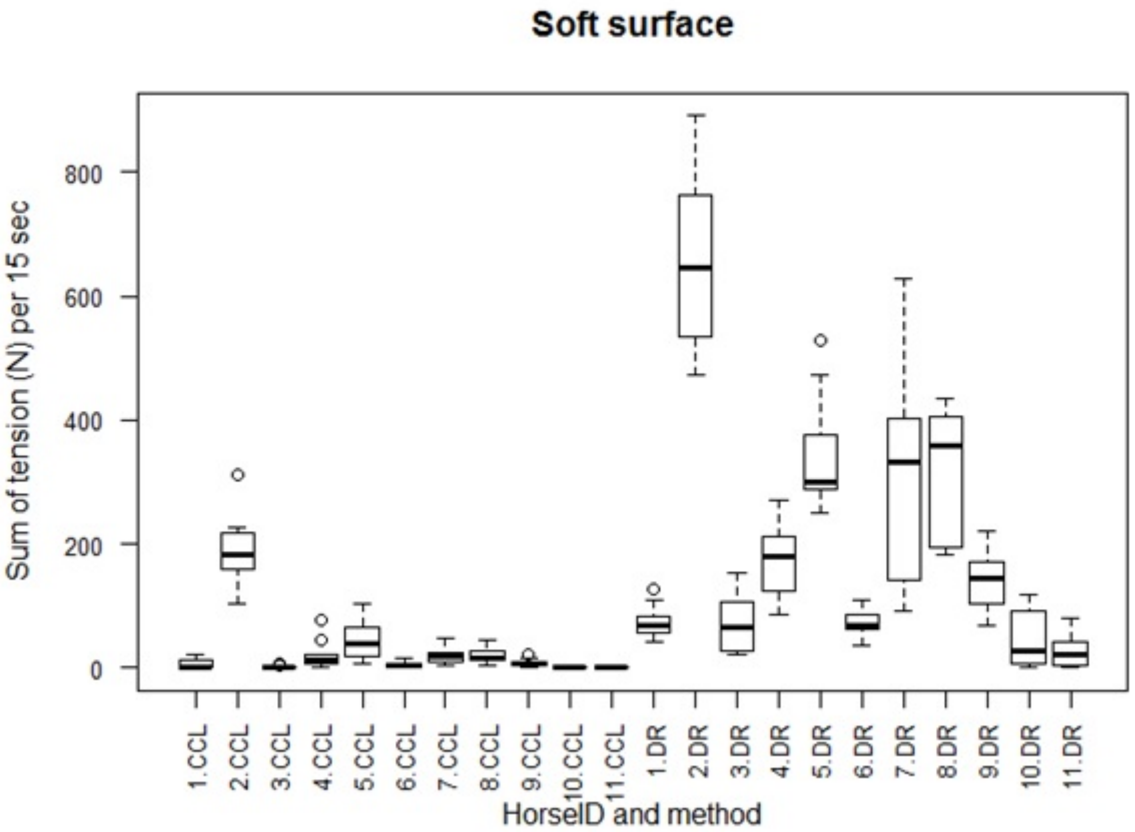

Supplement: Supplementary file 7 — Supplementary Item 7: Summary data. [file EVJ-50-825-s007.pdf]
