# Supplementary material for: The use of a rein tension device to compare different training methods for neck flexion in base‐level trained Warmblood horses at the walk
Source: Equine Vet J. 2018 Apr 6;50(6):825–30. doi: 10.1111/evj.12831 (PMC6174990; doi:10.1111/evj.12831)
Supplement: Supplementary file 8 — Supplementary Item 8: Rein tension per horse: Concord Leader Hard Surface Left Rein. [file EVJ-50-825-s008.pdf]

Equine Veterinary Journal

Supplementary Item 8: Rein tension per horse: Concord Leader Hard Surface Left Rein.

		CCL Hard Surface Left Rein						
		Minimum	Percentile 25	Median	Mean	Percentile 75	Maximum	% 0N
Number of the Horse	1	0	0	0	0	0	3	99.2
	2	0	0	0	0	0	8	85.0
	3	0	0	0	0	0	2	99.3
	4	0	0	0	0	0	5	92.9
	5	0	0	0	0	0	5	89.7
	6	0	0	0	0	0	4	97.8
	7	0	0	0	0	0	6	79.9
	8	0	0	0	0	0	4	91.8
	9	0	0	0	0	0	7	88.1
	10	0	0	0	0	0	2	99.4
	11	0	0	0	0	0	1	99.9

% 0N = percentage 0 Newton.
