# Supplementary material for: The use of a rein tension device to compare different training methods for neck flexion in base‐level trained Warmblood horses at the walk
Source: Equine Vet J. 2018 Apr 6;50(6):825–30. doi: 10.1111/evj.12831 (PMC6174990; doi:10.1111/evj.12831)
Supplement: Supplementary file 9 — Supplementary Item 9: Rein tension per horse: Concord Leader Hard Surface Right Rein. [file EVJ-50-825-s009.pdf]

Supplementary Item 9: Rein tension per horse: Concord Leader Hard Surface Right Rein.

		CCL Hard Surface Right Rein						
		Minimum	Percentile 25	Median	Mean	Percentile 75	Maximum	% 0N
Number of the Horse	1	0	0	0	0	0	2	98.7
	2	0	0	0	1	0	11	77.8
	3	0	0	0	0	0	1	99.3
	4	0	0	0	0	0	7	85.4
	5	0	0	0	0	0	7	79.0
	6	0	0	0	0	0	7	92.0
	7	0	0	0	1	0	7	76.3
	8	0	0	0	0	0	8	89.6
	9	0	0	0	0	0	7	86.0
	10	0	0	0	0	0	2	99.3
	11	0	0	0	0	0	0	100

% 0N = percentage 0 Newton.
