# Supplementary material for: The use of a rein tension device to compare different training methods for neck flexion in base‐level trained Warmblood horses at the walk
Source: Equine Vet J. 2018 Apr 6;50(6):825–30. doi: 10.1111/evj.12831 (PMC6174990; doi:10.1111/evj.12831)
Supplement: Supplementary file 11 — Supplementary Item 11: Rein tension per horse: Concord Leader Soft Surface Right Rein. [file EVJ-50-825-s011.pdf]

Equine Veterinary Journal

Supplementary Item 11: Rein tension per horse: Concord Leader Soft Surface Right Rein.

		CCL Soft Surface Right Rein						
		Minimum	Percentile 25	Median	Mean	Percentile 75	Maximum	% 0N
Number of the Horse	1	0	0	0	0	0	6	97.0
	2	0	0	0	1	1	16	62.1
	3	0	0	0	0	0	2	99.8
	4	0	0	0	0	0	9	87.7
	5	0	0	0	0	0	8	78.4
	6	0	0	0	0	0	5	97.6
	7	0	0	0	0	0	5	88.0
	8	0	0	0	0	0	9	87.9
	9	0	0	0	0	0	3	94.6
	10	0	0	0	0	0	1	99.7
	11	0	0	0	0	0	1	99.8

% 0N = percentage 0 Newton
