# Supplementary material for: The use of a rein tension device to compare different training methods for neck flexion in base‐level trained Warmblood horses at the walk
Source: Equine Vet J. 2018 Apr 6;50(6):825–30. doi: 10.1111/evj.12831 (PMC6174990; doi:10.1111/evj.12831)
Supplement: Supplementary file 12 — Supplementary Item 12: Rein tension per horse: Draw Reins Hard Surface Left Rein. [file EVJ-50-825-s012.pdf]

Equine Veterinary Journal

Supplementary Item 12: Rein tension per horse: Draw Reins Hard Surface Left Rein.

		Draw Reins Hard Surface Left Rein						
		Minimum	Percentile 25	Median	Mean	Percentile 75	Maximum	% 0N
Number of the Horse	1	0	0	0	1	1	15	58.3
	2	0	2	4	5	8	20	5.6
	3	0	0	0	0	0	7	82.9
	4	0	1	2	2	3	14	18.9
	5	0	1	2	3	4	16	12.7
	6	0	0	0	0	0	17	81.7
	7	0	1	2	3	4	20	16.4
	8	0	0	1	1	2	13	34.7
	9	0	0	0	1	1	16	52.9
	10	0	0	0	0	0	13	88.2
	11	0	0	0	0	0	4	85.3

% 0N = percentage 0 Newton
