# Supplementary material for: The use of a rein tension device to compare different training methods for neck flexion in base‐level trained Warmblood horses at the walk
Source: Equine Vet J. 2018 Apr 6;50(6):825–30. doi: 10.1111/evj.12831 (PMC6174990; doi:10.1111/evj.12831)
Supplement: Supplementary file 13 — Supplementary Item 13: Rein tension per horse: Draw Reins Hard Surface Right Rein. [file EVJ-50-825-s013.pdf]

Equine Veterinary Journal

Supplementary Item 13: Rein tension per horse: Draw Reins Hard Surface Right Rein.

		Draw Reins Hard Surface Right Rein						
		Minimum	Percentile 25	Median	Mean	Percentile 75	Maximum	% 0N
Number of the Horse	1	0	0	0	0	1	6	71.8
	2	0	1	3	4	6	24	8.1
	3	0	0	1	1	1	6	33.2
	4	0	1	1	2	2	21	17.4
	5	0	0	1	2	3	19	29.7
	6	0	0	0	0	0	20	83.1
	7	0	0	1	2	2	12	31.2
	8	0	1	2	2	3	16	35.4
	9	0	0	1	1	1	17	49.1
	10	0	0	0	1	1	34	64.0
	11	0	0	0	0	0	1	93.6

% 0N = percentage 0 Newton
