# Supplementary material for: The use of a rein tension device to compare different training methods for neck flexion in base‐level trained Warmblood horses at the walk
Source: Equine Vet J. 2018 Apr 6;50(6):825–30. doi: 10.1111/evj.12831 (PMC6174990; doi:10.1111/evj.12831)
Supplement: Supplementary file 14 — Supplementary Item 14: Rein tension per horse: Draw Reins Soft Surface Left Rein. [file EVJ-50-825-s014.pdf]

Supplementary Item 14: Rein tension per horse: Draw Reins Soft Surface Left Rein.

		Draw Reins Soft Surface Left Rein						
		Minimum	Percentile 25	Median	Mean	Percentile 75	Maximum	% 0N
Number of the Horse	1	0	0	0	1	1	16	72.9
	2	0	2	4	5	6	23	4.7
	3	0	0	0	0	0	45	85.0
	4	0	0	1	1	2	16	47.8
	5	0	1	2	2	3	16	14.6
	6	0	0	0	0	1	8	74.2
	7	0	1	2	3	4	17	19.8
	8	0	0	1	2	2	20	38.3
	9	0	0	0	1	1	22	50.6
	10	0	0	0	0	0	9	96.3
	11	0	0	0	0	0	6	77.9

% 0N = percentage 0 Newton.
