# Supplementary material for: The use of a rein tension device to compare different training methods for neck flexion in base‐level trained Warmblood horses at the walk
Source: Equine Vet J. 2018 Apr 6;50(6):825–30. doi: 10.1111/evj.12831 (PMC6174990; doi:10.1111/evj.12831)
Supplement: Supplementary file 15 — Supplementary Item 15: Rein tension per horse: Draw Reins Soft Surface Right Rein. [file EVJ-50-825-s015.pdf]

Supplementary Item 15: Rein tension per horse. Draw Reins Soft Surface Right Rein.

		Draw Reins Soft Surface Right Rein						
		Minimum	Percentile 25	Median	Mean	Percentile 75	Maximum	% 0N
Number of the Horse	1	0	0	0	0	1	11	73.3
	2	0	1	3	4	6	22	2.0
	3	0	0	1	1	1	47	43.6
	4	0	0	0	1	1	16	51.0
	5	0	1	2	2	3	17	20.6
	6	0	0	0	1	0	14	76.2
	7	0	0	1	1	2	12	46.8
	8	0	1	2	3	3	21	2.6
	9	0	0	0	1	1	14	60.8
	10	0	0	0	1	0	26	78.2
	11	0	0	0	0	0	3	96.8

% 0N = percentage 0 Newton.
