# Supplementary material for: The use of a rein tension device to compare different training methods for neck flexion in base‐level trained Warmblood horses at the walk
Source: Equine Vet J. 2018 Apr 6;50(6):825–30. doi: 10.1111/evj.12831 (PMC6174990; doi:10.1111/evj.12831)
Supplement: Supplementary file 16 [file EVJ-50-825-s016.docx]

Supplementary item 8. Descriptive statistical overview of rein tension per horse in condition Concord Leader Hard Surface Left Rein. % 0N = percentage 0 Newton.

|  | | CCL Hard Surface Left Rein | | | | | | |
| --- | --- | --- | --- | --- | --- | --- | --- | --- |
|  |  | Minimum | Percentile 25 | Median | Mean | Percentile 75 | Maximum | % 0N |
| Number of the Horse | 1 | 0 | 0 | 0 | 0 | 0 | 3 | 99.2 |
|  | 2 | 0 | 0 | 0 | 0 | 0 | 8 | 85.0 |
|  | 3 | 0 | 0 | 0 | 0 | 0 | 2 | 99.3 |
|  | 4 | 0 | 0 | 0 | 0 | 0 | 5 | 92.9 |
|  | 5 | 0 | 0 | 0 | 0 | 0 | 5 | 89.7 |
|  | 6 | 0 | 0 | 0 | 0 | 0 | 4 | 97.8 |
|  | 7 | 0 | 0 | 0 | 0 | 0 | 6 | 79.9 |
|  | 8 | 0 | 0 | 0 | 0 | 0 | 4 | 91.8 |
|  | 9 | 0 | 0 | 0 | 0 | 0 | 7 | 88.1 |
|  | 10 | 0 | 0 | 0 | 0 | 0 | 2 | 99.4 |
|  | 11 | 0 | 0 | 0 | 0 | 0 | 1 | 99.9 |

Supplementary item 9. Descriptive statistical overview of rein tension per horse in condition Concord Leader Hard Surface Right Rein. % 0N = percentage 0 Newton.

|  | | CCL Hard Surface Right Rein | | | | | | |
| --- | --- | --- | --- | --- | --- | --- | --- | --- |
|  |  | Minimum | Percentile 25 | Median | Mean | Percentile 75 | Maximum | % 0N |
| Number of the Horse | 1 | 0 | 0 | 0 | 0 | 0 | 2 | 98.7 |
|  | 2 | 0 | 0 | 0 | 1 | 0 | 11 | 77.8 |
|  | 3 | 0 | 0 | 0 | 0 | 0 | 1 | 99.3 |
|  | 4 | 0 | 0 | 0 | 0 | 0 | 7 | 85.4 |
|  | 5 | 0 | 0 | 0 | 0 | 0 | 7 | 79.0 |
|  | 6 | 0 | 0 | 0 | 0 | 0 | 7 | 92.0 |
|  | 7 | 0 | 0 | 0 | 1 | 0 | 7 | 76.3 |
|  | 8 | 0 | 0 | 0 | 0 | 0 | 8 | 89.6 |
|  | 9 | 0 | 0 | 0 | 0 | 0 | 7 | 86.0 |
|  | 10 | 0 | 0 | 0 | 0 | 0 | 2 | 99.3 |
|  | 11 | 0 | 0 | 0 | 0 | 0 | 0 | 100 |

Supplementary item 10. Descriptive statistical overview of rein tension per horse in condition Concord Leader Soft Surface Left Rein. % 0N = percentage 0 Newton.

|  | | CCL Soft Surface Left Rein | | | | | | |
| --- | --- | --- | --- | --- | --- | --- | --- | --- |
|  |  | Minimum | Percentile 25 | Median | Mean | Percentile 75 | Maximum | % 0N |
| Number of the Horse | 1 | 0 | 0 | 0 | 0 | 0 | 3 | 97.1 |
|  | 2 | 0 | 0 | 0 | 1 | 2 | 9 | 57.3 |
|  | 3 | 0 | 0 | 0 | 0 | 0 | 2 | 99.4 |
|  | 4 | 0 | 0 | 0 | 0 | 0 | 2 | 94.9 |
|  | 5 | 0 | 0 | 0 | 0 | 0 | 5 | 90.1 |
|  | 6 | 0 | 0 | 0 | 0 | 0 | 8 | 98.4 |
|  | 7 | 0 | 0 | 0 | 0 | 0 | 4 | 94.7 |
|  | 8 | 0 | 0 | 0 | 0 | 0 | 5 | 94.3 |
|  | 9 | 0 | 0 | 0 | 0 | 0 | 2 | 97.7 |
|  | 10 | 0 | 0 | 0 | 0 | 0 | 0 | 100 |
|  | 11 | 0 | 0 | 0 | 0 | 0 | 1 | 99.4 |

Supplementary item 11. Descriptive statistical overview of rein tension per horse in condition Concord Leader Soft Surface Right Rein. % 0N = percentage 0 Newton.

|  | | CCL Soft Surface Right Rein | | | | | | |
| --- | --- | --- | --- | --- | --- | --- | --- | --- |
|  |  | Minimum | Percentile 25 | Median | Mean | Percentile 75 | Maximum | % 0N |
| Number of the Horse | 1 | 0 | 0 | 0 | 0 | 0 | 6 | 97.0 |
|  | 2 | 0 | 0 | 0 | 1 | 1 | 16 | 62.1 |
|  | 3 | 0 | 0 | 0 | 0 | 0 | 2 | 99.8 |
|  | 4 | 0 | 0 | 0 | 0 | 0 | 9 | 87.7 |
|  | 5 | 0 | 0 | 0 | 0 | 0 | 8 | 78.4 |
|  | 6 | 0 | 0 | 0 | 0 | 0 | 5 | 97.6 |
|  | 7 | 0 | 0 | 0 | 0 | 0 | 5 | 88.0 |
|  | 8 | 0 | 0 | 0 | 0 | 0 | 9 | 87.9 |
|  | 9 | 0 | 0 | 0 | 0 | 0 | 3 | 94.6 |
|  | 10 | 0 | 0 | 0 | 0 | 0 | 1 | 99.7 |
|  | 11 | 0 | 0 | 0 | 0 | 0 | 1 | 99.8 |

Supplementary item 12. Descriptive statistical overview of rein tension per horse in condition Draw Reins Hard Surface Left Rein. % 0N = percentage 0 Newton.

|  | | Draw Reins Hard Surface Left Rein | | | | | | |
| --- | --- | --- | --- | --- | --- | --- | --- | --- |
|  |  | Minimum | Percentile 25 | Median | Mean | Percentile 75 | Maximum | % 0N |
| Number of the Horse | 1 | 0 | 0 | 0 | 1 | 1 | 15 | 58.3 |
|  | 2 | 0 | 2 | 4 | 5 | 8 | 20 | 5.6 |
|  | 3 | 0 | 0 | 0 | 0 | 0 | 7 | 82.9 |
|  | 4 | 0 | 1 | 2 | 2 | 3 | 14 | 18.9 |
|  | 5 | 0 | 1 | 2 | 3 | 4 | 16 | 12.7 |
|  | 6 | 0 | 0 | 0 | 0 | 0 | 17 | 81.7 |
|  | 7 | 0 | 1 | 2 | 3 | 4 | 20 | 16.4 |
|  | 8 | 0 | 0 | 1 | 1 | 2 | 13 | 34.7 |
|  | 9 | 0 | 0 | 0 | 1 | 1 | 16 | 52.9 |
|  | 10 | 0 | 0 | 0 | 0 | 0 | 13 | 88.2 |
|  | 11 | 0 | 0 | 0 | 0 | 0 | 4 | 85.3 |

Supplementary item 13. Descriptive statistical overview of rein tension per horse in condition Draw Reins Hard Surface Right Rein. % 0N = percentage 0 Newton.

|  | | Draw Reins Hard Surface Right Rein | | | | | | |
| --- | --- | --- | --- | --- | --- | --- | --- | --- |
|  |  | Minimum | Percentile 25 | Median | Mean | Percentile 75 | Maximum | % 0N |
| Number of the Horse | 1 | 0 | 0 | 0 | 0 | 1 | 6 | 71.8 |
|  | 2 | 0 | 1 | 3 | 4 | 6 | 24 | 8.1 |
|  | 3 | 0 | 0 | 1 | 1 | 1 | 6 | 33.2 |
|  | 4 | 0 | 1 | 1 | 2 | 2 | 21 | 17.4 |
|  | 5 | 0 | 0 | 1 | 2 | 3 | 19 | 29.7 |
|  | 6 | 0 | 0 | 0 | 0 | 0 | 20 | 83.1 |
|  | 7 | 0 | 0 | 1 | 2 | 2 | 12 | 31.2 |
|  | 8 | 0 | 1 | 2 | 2 | 3 | 16 | 35.4 |
|  | 9 | 0 | 0 | 1 | 1 | 1 | 17 | 49.1 |
|  | 10 | 0 | 0 | 0 | 1 | 1 | 34 | 64.0 |
|  | 11 | 0 | 0 | 0 | 0 | 0 | 1 | 93.6 |

Supplementary item 14. Descriptive statistical overview of rein tension per horse in condition Draw Reins Soft Surface Left Rein. % 0N = percentage 0 Newton.

|  | | Draw Reins Soft Surface Left Rein | | | | | | |
| --- | --- | --- | --- | --- | --- | --- | --- | --- |
|  |  | Minimum | Percentile 25 | Median | Mean | Percentile 75 | Maximum | % 0N |
| Number of the Horse | 1 | 0 | 0 | 0 | 1 | 1 | 16 | 72.9 |
|  | 2 | 0 | 2 | 4 | 5 | 6 | 23 | 4.7 |
|  | 3 | 0 | 0 | 0 | 0 | 0 | 45 | 85.0 |
|  | 4 | 0 | 0 | 1 | 1 | 2 | 16 | 47.8 |
|  | 5 | 0 | 1 | 2 | 2 | 3 | 16 | 14.6 |
|  | 6 | 0 | 0 | 0 | 0 | 1 | 8 | 74.2 |
|  | 7 | 0 | 1 | 2 | 3 | 4 | 17 | 19.8 |
|  | 8 | 0 | 0 | 1 | 2 | 2 | 20 | 38.3 |
|  | 9 | 0 | 0 | 0 | 1 | 1 | 22 | 50.6 |
|  | 10 | 0 | 0 | 0 | 0 | 0 | 9 | 96.3 |
|  | 11 | 0 | 0 | 0 | 0 | 0 | 6 | 77.9 |

Supplementary item 15. Descriptive statistical overview of rein tension per horse in condition Draw Reins Soft Surface Right Rein. % 0N = percentage 0 Newton.

|  | | Draw Reins Soft Surface Right Rein | | | | | | |
| --- | --- | --- | --- | --- | --- | --- | --- | --- |
|  |  | Minimum | Percentile 25 | Median | Mean | Percentile 75 | Maximum | % 0N |
| Number of the Horse | 1 | 0 | 0 | 0 | 0 | 1 | 11 | 73.3 |
|  | 2 | 0 | 1 | 3 | 4 | 6 | 22 | 2.0 |
|  | 3 | 0 | 0 | 1 | 1 | 1 | 47 | 43.6 |
|  | 4 | 0 | 0 | 0 | 1 | 1 | 16 | 51.0 |
|  | 5 | 0 | 1 | 2 | 2 | 3 | 17 | 20.6 |
|  | 6 | 0 | 0 | 0 | 1 | 0 | 14 | 76.2 |
|  | 7 | 0 | 0 | 1 | 1 | 2 | 12 | 46.8 |
|  | 8 | 0 | 1 | 2 | 3 | 3 | 21 | 2.6 |
|  | 9 | 0 | 0 | 0 | 1 | 1 | 14 | 60.8 |
|  | 10 | 0 | 0 | 0 | 1 | 0 | 26 | 78.2 |
|  | 11 | 0 | 0 | 0 | 0 | 0 | 3 | 96.8 |
